# Explainable drug repurposing via path based knowledge graph completion

**DOI:** 10.1038/s41598-024-67163-x

**Published:** 2024-07-18

**Authors:** Ana Jiménez, María José Merino, Juan Parras, Santiago Zazo

**Affiliations:** Information Processing and Telecommunications Center, Universidad Politécnica de Madrid, ETSI Telecomunicación, Avda. Complutense, 30, 28040 Madrid, Spain

**Keywords:** Drug repurposing, Heterogeneous knowledge graphs, Knowledge graph completion, Interpretability, Hetionet, Rule-based link prediction, Computational biology and bioinformatics, Computational models, Data processing, Machine learning, Predictive medicine

## Abstract

Drug repurposing aims to find new therapeutic applications for existing drugs in the pharmaceutical market, leading to significant savings in time and cost. The use of artificial intelligence and knowledge graphs to propose repurposing candidates facilitates the process, as large amounts of data can be processed. However, it is important to pay attention to the explainability needed to validate the predictions. We propose a general architecture to understand several explainable methods for graph completion based on knowledge graphs and design our own architecture for drug repurposing. We present XG4Repo (eXplainable Graphs for Repurposing), a framework that takes advantage of the connectivity of any biomedical knowledge graph to link compounds to the diseases they can treat. Our method allows methapaths of different types and lengths, which are automatically generated and optimised based on data. XG4Repo focuses on providing meaningful explanations to the predictions, which are based on paths from compounds to diseases. These paths include nodes such as genes, pathways, side effects, or anatomies, so they provide information about the targets and other characteristics of the biomedical mechanism that link compounds and diseases. Paths make predictions interpretable for experts who can validate them and use them in further research on drug repurposing. We also describe three use cases where we analyse new uses for Epirubicin, Paclitaxel, and Predinisone and present the paths that support the predictions.

## Introduction

Drug discovery is a time-consuming and high-cost process that involves several stages to obtain the approval of the authorities of a new drug. It takes 10–15 years and requires between $\$500$ million and $\$2$ billion. Moreover, approximately 90% of drugs fail in the early stages of development and toxicity testing and even among drugs that pass these steps, most fail due to side effects or adverse problems^1,2^.

Due to these limitations, the identification of new applications for existing drugs is a time-effective and cost-effective alternative, which is called drug repurposing. It allows the increase in treatment options for existing diseases and provides faster treatment for emerging diseases^2,3^.

The increasing amount of data available in recent years has led to the use of machine learning approaches for drug repurposing. This research focusses on drug repurposing based on biological knowledge graph datasets. Knowledge graphs are a set of nodes and edges that represent the relations between nodes. In the case of biological knowledge graphs, the nodes can be of different types, such as genes, compounds, side effects, diseases, etc. The goal of drug repurposing based on knowledge graphs is to discover links of type “treats” between entities of type compounds and diseases.

### Related work

Drug repositioning is a complex task that includes several approaches. In the state-of-the-art drug repurposing based on knowledge graphs, the most extended methods are based on embeddings, which map the nodes to a low-dimensional representation that summarises their graph location and the structure of their neighbourhood. Models such as those developed in^4–12^ are based on embeddings.

In^4^, a model based on attention called MT-DTI is developed to identify drug target interactions using binding affinity scores. In^5^, CoV-KGE, a deep learning model is used to generate a biomedical network and then applied embedding methods to find drug target interactions for COVID-19. In^6^, the authors use embeddings, but also diffusion networks and proximity-based algorithms for drug repurposing. In^13^, geometric deep learning is used to obtain smoothed feature representations of drugs and diseases, and then attention techniques are applied to propose candidate drugs for a given disease.

These methods provide predictions, but do not include any information on why or how the prediction was made. In addition, most of these methods cannot capture multistep relations. Interpretability is very important in this field, so several models include different methods to explain the predictions. Most of them are based on paths that relate drugs and diseases through the nodes of the graph. These paths provide biological explanations for why the compound can treat a certain disease. They also use metapaths, which are sequences of types of nodes and relations, to obtain paths.

Some methods use a small set of metapaths selected by an expert to obtain paths used for prediction^14–17^. The predictions are always based on the metapaths that are known to be useful. Other methods do the opposite, evaluating every possible metapath^18,19^ that connects the compound to the disease. In the first case, the model is based on expert knowledge rather than data, and the second approach is computationally expensive.

Other methods use tools for graph analysis such as^20,21^. In^21^, NeDRex is proposed as a platform for identifying subgraphs that represent the mechanisms of action of diseases that include genes and proteins, called disease modules. Then, disease modules are used to predict a list of drug candidates to treat a certain disease. They generated disease modules using network-based medical algorithms. However, the entities of the network and the relations between them are limited.

In^22^, a framework for drug repurposing named Torchdrug is developed that includes several important tasks for drug discovery, such as biomedical knowledge graph reasoning. In this field, they provided benchmarks for embedding-based models for Hetionet. However, they evaluate the complete graph and do not focus on repurposing, which is what we do in this work.

Other researches develop hybrid architectures that make predictions based on embeddings or other non-interpretable methods and apply some technique to provide explanations^23–26^. In^15^, KGML-xDTD is developed to predict repurposing candidates using embedding methods and random forest. Then, the model includes an actor-critic reinforcement learning approach to find paths between the drugs and the diseases that could explain the predictions. The agent was guided by demonstration paths, which are paths that can explain why a drug treats a disease. These paths were previously selected by experts.

KR4SL is presented in^27^ where they use a knowledge graph to learn semantic representations of gene pairs that encode the information of relational digraphs using an encoder-decoder framework. They use language models to enrich the semantics of KG for reasoning. They also use attention mechanisms to identify important subgraphs as explanations. This model is used to predict synthetic lethality partners for a primary gene.

MINERVA^28^ is a reinforcement learning agent used for general link prediction. PoLo^29^ is a modification of MINERVA which integrates the use of predefined rules for the task of drug repurposing.

### Our contribution: XG4Repo

In this work we address the drug repurposing problem using knowledge graphs, and we propose XG4Repo (eXplainable Graphs for Repurposing), which achieves good performance as well as high quality explanations. We focus on the interpretability and limitations of the models found in the literature to design our framework. Our main contributions are:

- Present a general architecture for knowledge graph drug repurposing and show how several algorithms fit this description. These models follow different approaches, but we show that they all share similar principles.
- Design XG4Repo, our own drug repurposing strategy that focusses on interpretability. Our approach combines and optimises state-of-the-art algorithms for graph completion and presents the results in natural language so they can be easily understood for humans. We provide a ready-to-use framework to propose candidates for repurposing. Our proposal is able to find high-quality paths with an adjustable computational cost, and works with any heterogeneous graph. Our method allows methapaths of different types and lengths, which are automatically generated and optimised based on data.
- We validate our approach by presenting three use cases in which we show that the predictions are interpretable and reliable, in line with the state-of-the-art in current clinical studies.

This tool is useful for experts in drug repurposing interested in starting a research process with a new drug. Instead of identifying potential disease candidates by hand, XG4Repo provides an ordered list as well as an explanation of why the disease can be treated by that drug. It allows processing large amounts of information contained in the knowledge graph in a short time. These predictions are then validated by the expert before starting the research.

## Methods

### Knowledge graph drug repurposing background

Graphs are collections of objects (nodes) and the set of interactions (edges) between pairs of these objects^30^. Knowledge graphs ($\mathscr {G}$) are a particular type of multirelational graph where the information is defined by a set of existing triples, including a head node (*h*), a tail node ($t^*$) and a relation (*r*) that links them:

1 $$\begin{aligned} (h,r,t^*) \in \mathscr {G} \end{aligned}$$

Drug repurposing on knowledge graphs can be seen as a task of link prediction, where we ask the graph which diseases a certain compound treats. We can understand the problem as a query that has to be solved by the graph. The query is composed of a “compound” *c* as the head, and the relation “treats”. The answer to this query is a disease *d* that can be treated with the compound, which is the tail of the triple.

2 $$\begin{aligned} (c,treats,d^*) \in \mathscr {G} \end{aligned}$$

The problem can be formulated in terms of the probability of success of the compound over the disease *d*, where the objective is that the answer equals the tail of the triple $d=d^*$, and which is conditioned on the existing graph:

3 $$\begin{aligned} p(c,treats,d=d^*\mid \mathscr {G})= p(d\mid \mathscr {G},(c,treats))= p(d\mid \mathscr {G},q) \end{aligned}$$

where $q=(c,treats)$ is the query.

### Path-based drug repurposing

Path-based drug repurposing leverages the connectivity of the graph to predict the disease that can be treated with a certain drug and also to provide a biological explanation of the prediction. These methods provide paths, which are sequences of nodes and relations that start and the head node of the query, in this case the compound, and follow different relations and nodes to arrive at the candidate disease. Another important concept is the metapath, which is a sequence of types of nodes and types of relations. For example, in the path (Epirubicin ${\mathop {\longrightarrow }\limits ^{\text { upregulates } }}$ Gene EGF ${\mathop {\longrightarrow }\limits ^{\text { regulates } }}$ Gene BRAF ${\mathop {\longrightarrow }\limits ^{\text { is associated to } }}$ Breast cancer) is a particularisation of the metapath (Compound ${\mathop {\longrightarrow }\limits ^{\text { upregulates } }}$ Gene ${\mathop {\longrightarrow }\limits ^{\text { regulates } }}$ Gene ${\mathop {\longrightarrow }\limits ^{\text { is associated to } }}$ Disease). Metapaths are also called rules in certain contexts.

To generate paths, a strategy is needed. This strategy can be represented by a policy $\mu$, which indicates the node that should follow the current node on the path. Another method of obtaining paths is the use of rules *z* or metapaths that applied to the graph generate paths. This strategy conditionally characterises the probability of a cadidate disease as:

4 $$\begin{aligned} p(d\mid \mathscr {G},q,\mu ) \end{aligned}$$

in the case of policies, and

5 $$\begin{aligned} p(d\mid \mathscr {G},q,z) \end{aligned}$$

in the case of rules.

The main reason to use paths is that predictions can be interpreted by healthcare professionals, which is necessary to validate candidate diseases for further research. Paths provide information about the side effects, targets, or anatomies involved in the biological mechanism.

We propose an architecture that generalises several methods for path-based graph completion, and we show the relation between them. We also present a mathematical formulation in Supplementary Data that unifies these models to understand them as a particularisation of the architecture described in this work.

**Figure 1:**
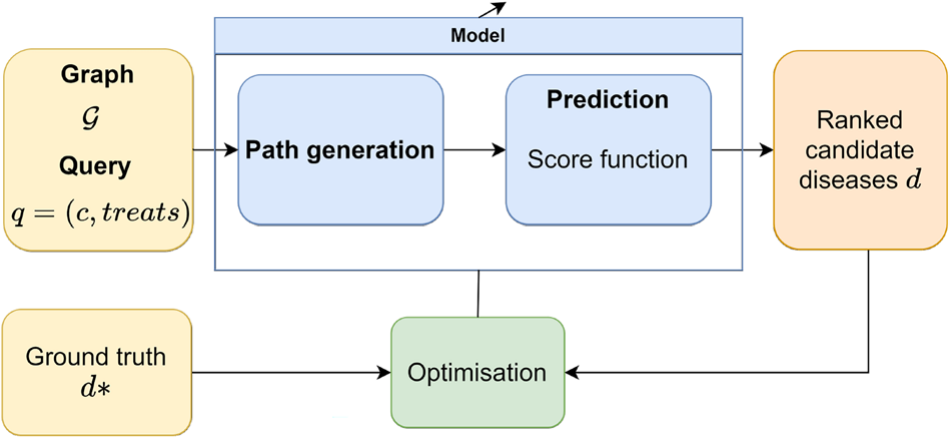
General architecture to train path-based drug repurposing models. The information is presented in the form of a knowledge graph composed of triples (in yellow). The input of the model is the graph and the query that has to be solved. The model (in blue) generates paths between compounds and different diseases. A score is computed to assess the quality of the paths and diseases that they propose. The model is optimised (green block) so that the ground truth diseases have the highest score.

The objective of the model is to predict diseases that can be treated by a certain compound using paths to connect the compound to the disease. Figure 1 shows the training process where the model is optimised to generate high-quality paths between the heads and tails of the queries. In the drug repurposing case, given a compound, the model generates paths that end in a set of candidate diseases. A score function evaluates the quality of the proposed diseases. The model learns to give high scores to diseases that are known to be treated with the compound. Therefore, other diseases that have high scores are good candidates for repurposing.

Taking into account the concepts of path generator and score function, the probability of the candidate for repurposing can be parameterised by $\theta$ and $\omega$.

6 $$\begin{aligned} p(d \mid \mathscr {G}, q) \rightarrow p_{\omega , \theta }(d \mid \mathscr {G}, q) \end{aligned}$$

where the path generation process is parameterised with $\theta$ and the score function with $\omega$.

This expression can be decomposed into two processes: path generation and reasoning prediction. The objective of the path generator is to obtain the paths from components to diseases, and the reasoning predictor uses those paths to answer queries. As explained before, paths can be generated using policies or rules.

7 $$\begin{aligned}&p_{\omega , \theta }(d \mid \mathscr {G}, q) = \sum _{\mu } p_\omega (d \mid \mathscr {G}, q, \mu )p_{\theta }(\mu \mid \mathscr {G},q) \end{aligned}$$

8 $$\begin{aligned}&p_{\omega , \theta }(d \mid \mathscr {G}, q)=\sum _{z} p_\omega (d \mid \mathscr {G}, q, z)p_{\theta }(z \mid \mathscr {G},q) \end{aligned}$$

Several models fit this description with minor modifications, as we will show in the next sections. There are different approaches, some models are based on rules, while others use reinforcement learning techniques. These approaches fit into the general model that we propose because they are based on similar ideas. Further details on this formulation can be found in Supplementary Data.

#### Fixed path generator

There are several ways to generate paths, and the simplest is to use a fixed path generator. We can generate paths using a fixed generator following different principles, for example, random walks. This is the approach followed in AnyBURL^31^. AnyBURL is a bottom-up technique for efficiently learning logical rules from large knowledge graphs inspired by classic bottom-up rule learning approaches. AnyBURL learns as many rules as possible by sampling random paths over a predetermined time interval. Then, each rule is evaluated according to the rate of correct positive predictions among all inferred predictions to obtain the confidence of the rule. The particularisation of the rules in the graph given a query generates paths between the compound and the candidate diseases. This is done in the path generator block in Fig. 1.

Several rules generate the same candidate, so an aggregation of the score of each rule is required to find the final score of a candidate. This corresponds to the prediction block in Fig. 1. There are three different approaches to determine the score of each candidate: Maximum score and Noisy-OR originally proposed with AnyBURL in^32^, and Non-redundant Noisy-OR proposed as a framework called SAFRAN^33^.

The optimisation in this case is very simple as the only task is to keep the rules that have a high enough confidence so that they can provide good predictions.

**Figure 2:**
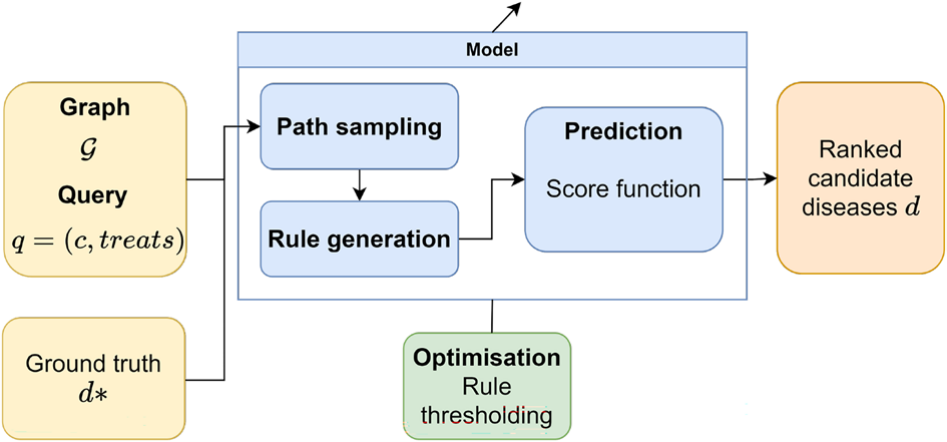
Description of AnyBURL based on our architecture. In this case, the input of the model is the whole graph (training set) including the ground truth. Paths are sampled based on random walk, and they are used to generate rules using a bottom-up approach. Then the confidence of the rule is computed, so only the rules with a confidence higher than a threshold are used for prediction. Rules are applied to the graph to obtain predictions which are ranked using the confidence of the rule.

#### Reinforcement learning based path generator

The next step is to use path generators that can be updated based on data to learn the best way to traverse the graph to make predictions. Some methods use reinforcement learning to model the trajectory on the graph as a Markov Decision Process. Starting from the head node, the agent learns to walk to the tail node, choosing intermediate nodes step by step, taking into account the path history. Paths are generated based on policy $\mu$, which is the strategy to traverse the graph to make good predictions.

This approach is followed in MINERVA^28^ and its variants^29,34^. In the drug repurposing context, the environment is the graph, and the possible actions are all the links the agent can choose from a certain node to the next. The objective of the agent is to move from a compound node to a disease node which is linked through the relations “treats”. The state includes all nodes and relations travelled through to the current node, so the next action depends on the whole path. Moreover, it is necessary to define a reward function $R\left( \pi ^n\mid q\right)$ that indicates whether the path $\pi ^n$ provides good predictions or not.

**Figure 3:**
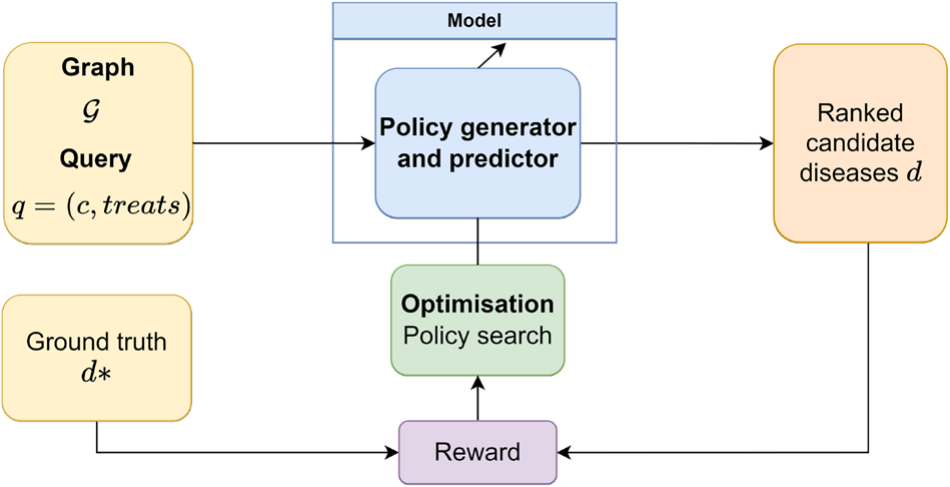
Description of MINERVA based on our architecture. The core of the algorithm is the policy generator, which is trained to obtain the best policy through the reward using policy search. Paths are sampled from the generator to obtain candidate diseases which are ranked according to the path that proposes them.

In this case, as shown in Fig. 3, the main element is a policy generator that is trained to maximise long-term reward. Paths are sampled from the generator to connect the compound to the candidate diseases. The prediction and calculation of the score function are included in this block, because the score is directly related to the policy followed to generate the path as shown in the Supplementary Data.

The long-term reward over the policy is defined as:

9 $$\begin{aligned} \mathbb {E}_{\pi ^n \sim \mu _\theta }\left[ R\left( \pi ^n\mid q\right) \right] \end{aligned}$$

where the policy generator is parameterised with $\theta$ and $\pi ^n$ represent the paths sampled from policy $\mu _\theta$. After some manipulation found in the Supplementary material, the function that needs to be optimised is the following:

10 $$\begin{aligned} \frac{1}{N}\sum _{n=1}^N R\left( \pi ^n\mid q\right) \log p_\theta (\pi ^n\mid \mathscr {G}, q) \end{aligned}$$

which averages the paths $\pi ^n$ sampled from policy $\mu _\theta$ weighted by the reward $R\left( \pi ^n\mid q\right)$ of the path. *N* is the number of paths.

PoLo^29^ is a modification of MINERVA that focusses on drug repurposing. The model includes a term in the reward related to how similar the path is to a set of manually crafted metapaths considered reliable for repurposing. This model relies on the existence of expert knowledge to improve the results of MINERVA.

#### Path generator using variational inference

Reasoning based on reinforcement learning has the problem that the action space is large and the reward is sparse, as few paths lead to the correct answer and a positive reward. For that reason, there are models that use rules as latent variables to make predictions. These rules (*z*) allow for the interpretability of the results and support the predictions. RNNLogic^35^ follows this approach.

**Figure 4:**
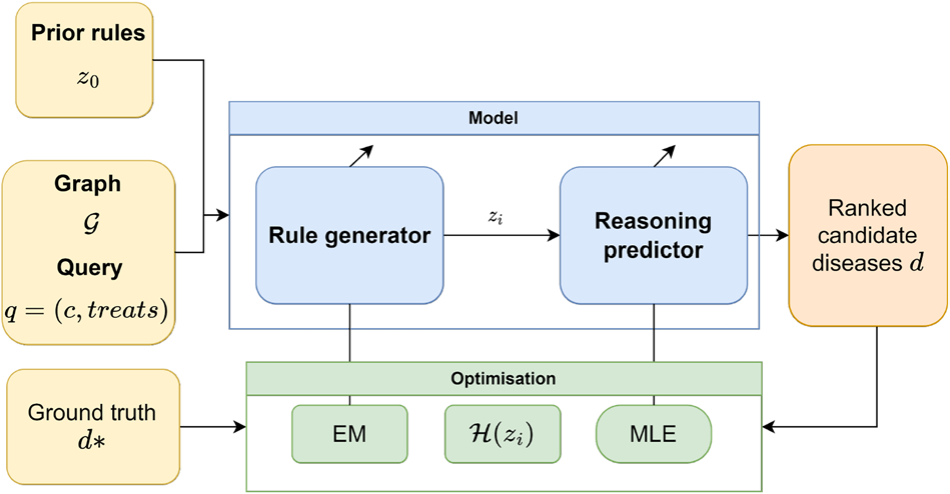
Description of RNNLogic based on our architecture. In addition to the graph and the query, there is another input which is a set of prior rules to initialize the generator. The model consists of a rule generator and a reasoning predictor. A set of rules is sampled and used for prediction. During training, the predictor is updated using maximum likelihood estimation (MLE). Combining information of the generation and the prediction, a score for each rule $\mathscr {H}(z_i)$ is computed and it is using during the training of the generator which is based on expectation maximisation.

The model includes a rule generator and a reasoning predictor that apply the rules to propose candidate answers for the query, as shown in Fig. 4. The rule generator returns a set of logic rules conditioned on the query, which are given to the reasoning predictor for query answering. The reasoning predictor computes the likelihood of the answer conditioned on the logic rules and the existing knowledge graph $\mathscr {G}$, $p_\omega (d\mid \mathscr {G}, q, z)$. At each training iteration, a few logic rules are sampled from the generator, which are fed into the reasoning predictor to try these rules for prediction. The distribution $p(d\mid \mathscr {G},q)$ can be calculated according to Eq. (7) as:

11 $$\begin{aligned} p_{w, \theta }(d \mid \mathscr {G}, q)=\sum _{z} p_w(d \mid \mathscr {G}, q, z) p_\theta (z \mid q) \end{aligned}$$

which is the objective function that has to be optimised by the whole model. This task is divided as the generator and predictor use different optimisation algorithms, but both contribute to a common goal.

The generator $p_\theta (z\mid q)$ is updated using expectation maximisation (EM), and the optimisation of the predictor $p_w(d \mid \mathscr {G}, q, z)$ is based on maximum likelihood principles (MLE). Given the graph and the query, a set of rules is sampled and then used for the prediction $\hat{z} \sim p_\theta (z \mid q)$. Based on the results of the predictions, a score is calculated for each rule $\mathscr {H}(\hat{z}_i)$. It includes information from both the generation and prediction processes, so it is possible to know which are the high-quality rules for prediction $\hat{z}_I$.

To optimise the generator $p_\theta (z \mid q)$, a set of high-quality rules $z_I$ is selected according to $\mathscr {H}(\hat{z}_I)$. For each data instance, the set of rules $\hat{z}_I$ is treated as part of the training data, and the generator is updated by maximising the logarithmic likelihood of $\hat{z}_I$. Moreover, $\mathscr {H}(\hat{z}_I)$ has information on the quality of the rules, so it can also be included in the generator optimisation in the form of weights of each rule:

12 $$\begin{aligned} \mathscr {H}(\hat{z}_I) \log p_\theta \left( \hat{z}_I \mid q\right) =\sum _{z_i \in \hat{z}_i} \mathscr {H}(\hat{z}_I) p_\theta \left( \hat{z}_i \mid q\right) \end{aligned}$$

The function to be maximised is the average of the rules weighted by the score of the rules.

Rules generate paths that end in candidate diseases which are ranked according to a score computed based on trainable parameters related to the importance of rules and paths. The score measures the reliability of the predictions and can be used in the drug repurposing case study to evaluate and interpret candidates for repurposing.

### XG4Repo

We have developed XG4Repo, a ready-to-use framework for computational drug repurposing using knowledge graphs. This framework is capable of predicting candidate diseases for repurposing and providing informative explanations to help a human expert in the research of new treatments.

Our proposal is a particularisation of the described architecture that combines state-of-the-art methods for graph completion and optimises them for drug repurposing. In Fig. 2 we see the architecture of XG4Repo. The core of the framework is RNNLogic, because it provides informative rules and achieves good results. To initialise the generator, we have used the rule miner in AnyBURL, as the generated rules are good for prediction tasks. These rules need to be processed to be readable by the generator and filtered to remove those that are not general enough.

**Figure 5:**
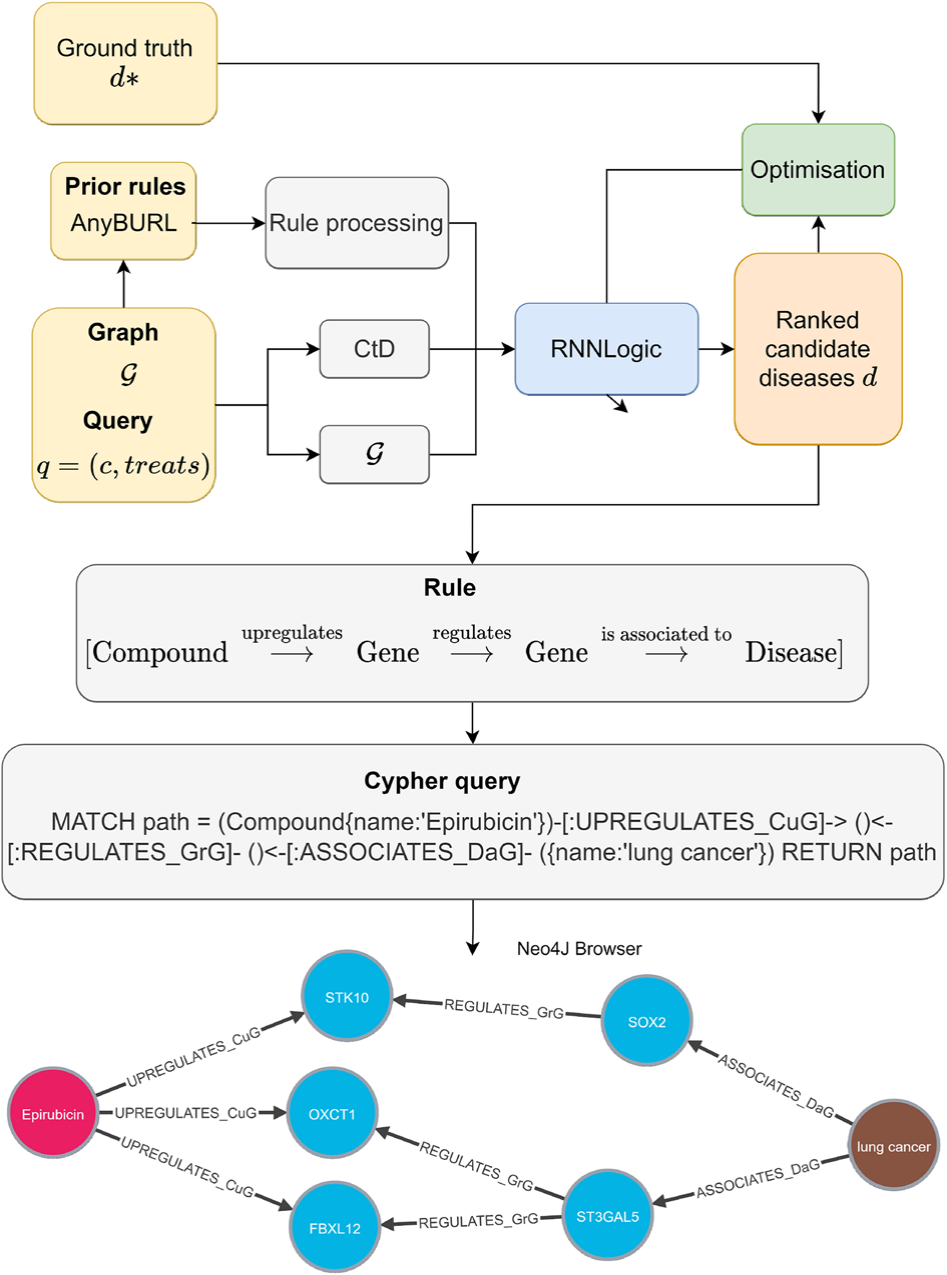
Description of XG4Repo architecture. The first step of the process is to generate a set of prior rules using AnyBURL rule miner. These rules are processed and used as priors in the generators. The model is trained using only the triples “compound treats disease” so the computational complexity is reduced. Once the predictions are made, the rules and corresponding scores are stored in natural language, so they can be easily understood. Moreover, our framework can generate Cypher queries to obtain the paths in Hetionet given the rules. This adds interpretability to the predictions without adding extra storage requirements.

We have adapted the graph completion task to repurposing. In conventional graph completion, the model is trained to predict queries that include every type of relation. In drug repurposing, we are only interested in the relation “treats”, so the model is specifically optimised to find diseases that can be treated by compounds. The training set only includes triples of “compound treats disease” but the whole graph can be traversed to find paths that include nodes and relations of any kind. Reducing the training set reduces computational time and resources, which is very interesting in the case of drug repurposing. As we see in Fig. 5, “compound treats disease” (CtD) triples are differentiated from the rest of the graph.

A key element in our design is a module for the interpretability of the results, where we can look for the predictions, the rules that support them, how important these rules are, and the paths that connect the compound to the disease. This is useful for those experts interested in the repurposing task, as they get predictions and explanations in natural language. Moreover, the code generates Cypher queries to obtain the paths generated by any specific rule on Hetionet. This is more efficient than storing every path generated by the rules. The code of XG4Repo is ready-to-use and available in https://github.com/AnaJimBej/XG4Repo.

An important aspect of our interpretability-based contribution is that the explainability module is integrated with the prediction process. It is not just a model to make predictions, it is a framework that starting from prior knowledge and a candidate drug predicts the diseases it can treat and the explanation of why it would work in natural language. This makes it possible for it to be used by end users who want to initiate drug repurposing research.

### Data

Hetionet^18^ was developed within the Rephetio project with the aim of creating a knowledge graph suitable for different tasks related to drug repurposing, and is publicly available. This database has been chosen as it is public and can therefore be used for comparison with other state-of-the-art methods. In addition, it has been used for similar tasks related to drug repurposing.

One notable aspect of Hetionet is its emphasis on incorporating multiple types of relations, such as drug-target interactions, gene-disease associations, and pathway connections. This comprehensive approach enables researchers to explore and prioritise potential drug repurposing opportunities, as well as gain insight into the underlying mechanisms of diseases.

Among the 2,250,197 triplets that make up the knowledge graph, only 755 correspond to “compound treats disease”. An 80-10-10% split was applied to divide the data set for training, testing and validation, respectively, obtaining the triplets. Of the 755 triplets of “compound treats disease”, 598 are used to train the model, 82 for testing, and the remaining 75 triplets are used for validation.

The model is specifically trained to predict “compound treats disease” relations. The rule generator learns the relations of every triplet in the graph. The rules generated to make the predictions include relations of all types, so the paths generated can include any of the nodes or relations that make up the graph.

The graph has been augmented with inverse relations, which, for each triplet, go from tail to head $(t, r^{-1}, h)$. This adds flexibility to the rules and allows more connections between the nodes.

### Evaluation metrics

Once a model has been trained, it has to be evaluated. The output of the model is a score for each possible answer for the test. During the test, we check that the disease of the triple being evaluated ($d^*$) receives a high score. Candidate diseases are ordered by decreasing score and the rank is defined as the position of the disease of the ground truth ($d^*$) in the list of candidates.

Based on the rank, several metrics are computed, which aggregate in a single number the performance of the model^36^. In this work, we have evaluated the models using mean reciprocal rank (MRR), Hits@1, Hits@3 and Hits@10.

The metrics calculated in this research are filtered as described in^36^. Moreover, binomial proportion confidence intervals are applied to compare the performance of the models as the size of the test set does not have enough samples to use the Gaussian approximation. It provides an interval estimate of a success probability *p* when only the number of experiments *n* and the number of successes $n_s$ are known.

## Results

We have trained several path-based graph completion models for Hetionet. The models being compared are XG4Repo, which represents our approach, MINERVA as a representation of reinforcement learning-based methods, and AnyBURL-based methods. For AnyBURL, we test three prediction strategies: Maximum score, Noisy-OR and SAFRAN. In the case of MINERVA and XG4Repo, we have trained only “drug treats disease” triples. For models based on AnyBURL, we have trained over every relation and then filtered test triples for evaluation, so test samples are the same in all cases. In Table 1, we present test metrics for models when the path length is set to three in all cases for comparison. For XG4Repo, 100 rules have been sampled from the generator.

**Table 1:** Comparison of the test results on “compound treats disease” on Hetionet using explainable methods.

Method	MRR	Hits@1	Hits@3	Hits@10
PoLo	0.402	0.314	0.428	0.609
PoLo (pruned)	0.430	0.337	0.47	0.641
AnyBURL(maxscore)	0.520	0.390	0.573	0.817
AnyBURL(noisy-OR)	0.511	0.366	0.598	0.805
SAFRAN	0.563	0.439	0.598	0.793
MINERVA	0.359	0.244	0.378	0.622
XG4Repo	0.612	0.488	0.671	0.890

We include the results reported by PoLo^29^ for comparison.

We include the results reported in PoLo^29^, because as far as we know, this is the only work that provides results for “compound treats disease” on Hetionet. We see that XG4Repo obtains better metrics under the same experimental conditions.

**Figure 6:**
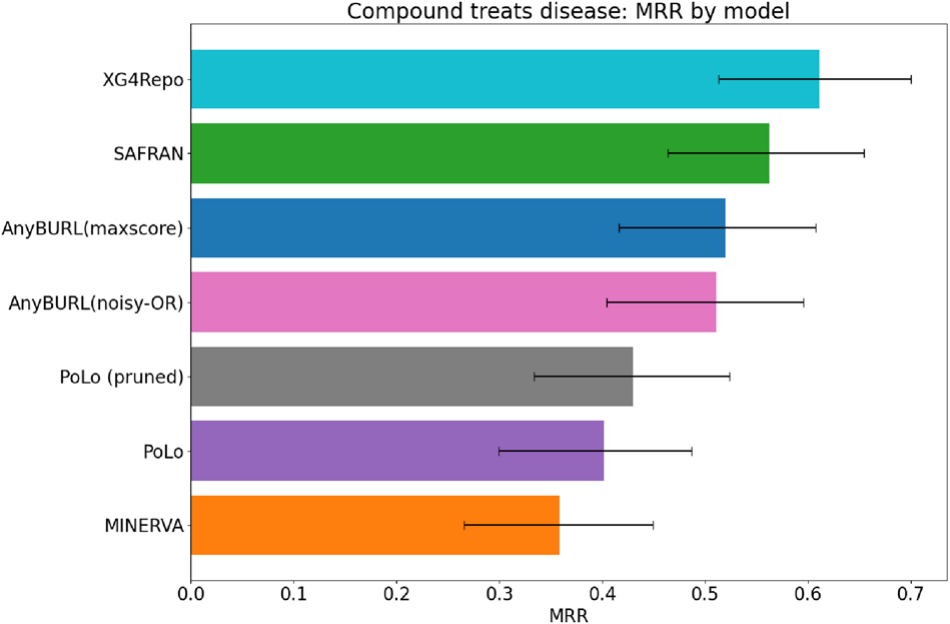
Comparison of the MRR of different models for “compound treats disease” in Hetionet. The confidence intervals at 90% are included. Rule-based models work better than reinforcement learning. Due to the small test set, confidence intervals for rule-based models overlap, so it is not possible to identify the best performing one in statistical terms.

In Fig. 6, we show the MRR of each model including $90\%$ confidence interval. As the test set only includes 82 triples, the confidence intervals are large, so for most models, they overlap. Confidence intervals can be expected to narrow in larger knowledge graphs. For that reason, we propose XG4Repo as a promising tool to propose repurposing candidates and to provide meaningful explanations about the predictions. We can see that XG4Repo is clearly better than models based on reinforcement learning models, as it can generate more variate paths that lead to different candidates. We show the performance of the model and the interpretability of the predictions using three use cases of repurposing.

### Use cases

In this section, we present three use cases of repurposing using the framework we have developed. The goal is to obtain diseases that can be treated with Epirubicin, Paclitaxel, and Prednisone using the methods explained previously. We include the predictions of AnyBURL-based models and MINERVA to support consistency in the predictions of our framework, as in most cases different models propose the same candidates.

The rules provided in this section are generated by XG4Repo and have a length of three steps. Paths provide explanations for the predictions and include the score by which the rule contributes to the prediction.

We also include some references to show that there are research and clinical trials that use drugs to treat diseases that have been proposed by the model. This shows that our framework can be a useful tool for healthcare professionals, as it is capable of handling larger amounts of data and coming to the same conclusions as them. It is an interesting first approximation for the processing of large datasets that has to be validated by further research.

#### Epirubicin

Epirubicin is a chemotherapy drug that is used to treat various types of cancer. Epirubicin treats 14 types of cancer according to Hetionet. The test set includes breast cancer, bone cancer, sarcoma, and uterine cancer, and the rest of the diseases are used for training.

In Table 2, we show the test results for different models and include the position of the disease in the prediction (rank). We see that the triples in the test set (in bold), those that we know to be true, are proposed as the first candidates for repurposing for every model except for MINERVA. Different models tend to provide similar predictions. The results of our model are consistent with the state-of-the-art in particular cases as shown in Table 2 and better for the whole graph as shown in Fig. 6.

**Table 2:** Top 10 diseases predicted by each model for the query “Epirubicin treats disease”.

Disease	AnyBURL maxscore	AnyBURL noisy-OR	SAFRAN	MINERVA	XG4Repo
**Breast cancer**	1	1	3	8, 9	1
Lung cancer^37^	2	2	2		2
**Sarcoma**	3	6	6		3
**Kidney cancer**	5	4	1		4
Muscle cancer^38^	4	5	5		5
**Bone cancer**	6	7	4		6
Melanoma^39^					7
Lymphatic system cancer^40^			8		8
Germ cell cancer^41^	9		10		9
Coronary artery disease					10
Hypertension		3			
Colon cancer^42^	7	10	7		
Multiple sclerosis	8	9	9		
Brain cancer^43^	10				
Asthma		8			
Epilepsy				1, 2, 5, 6, 10	
Osteoporosis				3	
Atopic dermatitis				4	

Each disease is predicted in a different position (rank) for different models. Diseases are ordered by XG4Repo results. Notice that in MINERVA, several paths can lead to the same node in different realisations. In bold, those diseases that are in the test set and, therefore, are true answers of the query.

##### Epirubicin treats breast cancer

All the models propose breast cancer as a candidate disease to be treated by Epirubicin. We already know that this prediction is true, as it is in the test set. The models can effectively identify those diseases that could improve with the use of the drug. In Table 3, we see the most important rules for this prediction according to XG4Repo and a path length of 3. These metapaths are expressive and include useful information about the targets of the disease. Most of them match the metapaths found relevant in^18^. The score of the rule shows the importance of the rule for prediction. This score is related to Eq. (12) and helps the human expert in drug repurposing interpret the prediction.

**Table 3:** Top rules for Epirubicin treats breast cancer and the corresponding scores.

Score	Rule
1793	[Compound ${\mathop {\longrightarrow }\limits ^{\text { causes } }}$ Side effect ${\mathop {\longrightarrow }\limits ^{\text { is caused by } }}$ Compound ${\mathop {\longrightarrow }\limits ^{\text { treats } }}$ Disease ]
1007	[Compound ${\mathop {\longrightarrow }\limits ^{\text { upregulates } }}$ Gene ${\mathop {\longrightarrow }\limits ^{\text { is expressed by } }}$ Anatomy ${\mathop {\longrightarrow }\limits ^{\text { is localized to } }}$ Disease ]
636	[Compound ${\mathop {\longrightarrow }\limits ^{\text { upregulates } }}$ Gene ${\mathop {\longrightarrow }\limits ^{\text { regulates } }}$ Gene ${\mathop {\longrightarrow }\limits ^{\text { is associated to } }}$ Disease ]
412	[Compound ${\mathop {\longrightarrow }\limits ^{\text { upregulates } }}$ Gene ${\mathop {\longrightarrow }\limits ^{\text { is upregulated by } }}$ Compound ${\mathop {\longrightarrow }\limits ^{\text { treats } }}$ Disease]
378	[Compound ${\mathop {\longrightarrow }\limits ^{\text { treats } }}$ Disease ${\mathop {\longrightarrow }\limits ^{\text { associates } }}$ Gene ${\mathop {\longrightarrow }\limits ^{\text { is associated to } }}$ Disease]
202	[Compound ${\mathop {\longrightarrow }\limits ^{\text { upregulates } }}$ Gene ${\mathop {\longrightarrow }\limits ^{\text { is downregulated by } }}$ Anatomy ${\mathop {\longrightarrow }\limits ^{\text { is localized to } }}$ Disease]
200	[Compound ${\mathop {\longrightarrow }\limits ^{\text { upregulates } }}$ Gene ${\mathop {\longrightarrow }\limits ^{\text { is upregulated by } }}$ Anatomy ${\mathop {\longrightarrow }\limits ^{\text { is localized to } }}$ Disease]

**Figure 7:**
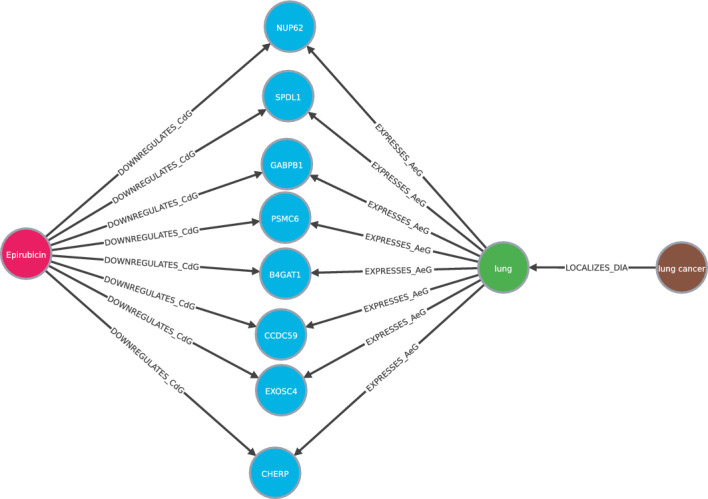
Set of paths that represent the triple Epirubicin treats breast cancer following the metapath [Compound ${\mathop {\longrightarrow }\limits ^{\text { upregulates } }}$ Gene ${\mathop {\longrightarrow }\limits ^{\text { is expressed by } }}$ Anatomy ${\mathop {\longrightarrow }\limits ^{\text { is localized to } }}$ Disease]. The number of nodes has been limited to facilitate visualization.

We do know that the relation treats exists between Epirubicin and breast cancer and have presented the rules that show the mechanisms that explain it. Moreover, we can see the paths to identify the nodes that relate the query and the prediction. In Fig. 7 we see some paths that follow the rule: [Compound ${\mathop {\longrightarrow }\limits ^{\text { upregulates } }}$ Gene ${\mathop {\longrightarrow }\limits ^{\text { is expressed by } }}$ Anatomy ${\mathop {\longrightarrow }\limits ^{\text { is localized to } }}$ Disease]. We also provide Cypher queries to explore all these paths in Neo4J browser along with the code.

##### Epirubicin treats lung cancer

**Figure 8:**
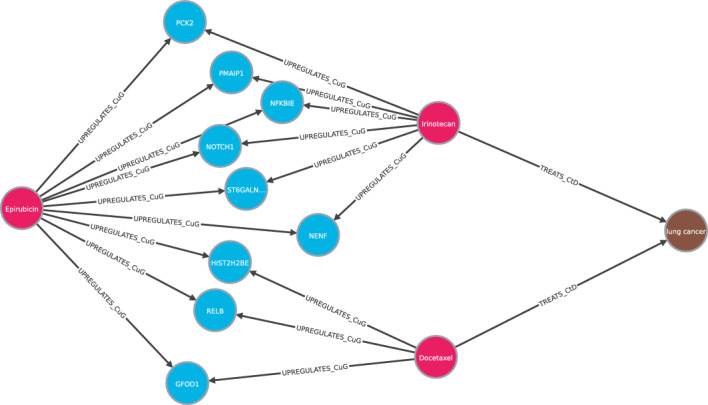
Set of paths that represent the triple “Epirubicin treats lung cancer” following the metapath [Compound ${\mathop {\longrightarrow }\limits ^{\text { upregulates } }}$ Gene ${\mathop {\longrightarrow }\limits ^{\text { is upregulated by } }}$ Compound ${\mathop {\longrightarrow }\limits ^{\text { treats } }}$ Disease]. The number of nodes has been limited to facilitate visualization.

Most models predict that lung cancer can be treated with Epirubicin in second position. This disease is not included in Hetionet, so it is a candidate. DrugBank^37^ is an online free-to-access database that contains information on drugs and drug targets. In^37^, DrugBank includes Epirubicin as treatment for Non-Small Cell Lung Carcinoma and Small Cell Lung Cancer (SCLC). Then, the models has been able to predict a treatment for a disease that healthcare community has accepted, even though it is not included in the knowledge graph used for training. We also include the most important rules for this prediction Table 4 and some paths in Fig. 8.

**Table 4:** Top rules for Epirubicin treats lung cancer and the corresponding scores.

Score	Rule
1208	[Compound ${\mathop {\longrightarrow }\limits ^{\text { causes } }}$ Side effect ${\mathop {\longrightarrow }\limits ^{\text { is caused by } }}$ Compound ${\mathop {\longrightarrow }\limits ^{\text { treats } }}$ Disease]
713	[Compound ${\mathop {\longrightarrow }\limits ^{\text { upregulates } }}$ Gene ${\mathop {\longrightarrow }\limits ^{\text { is expressed by } }}$ Anatomy ${\mathop {\longrightarrow }\limits ^{\text { is localized to } }}$ Disease]
340	[Compound ${\mathop {\longrightarrow }\limits ^{\text { upregulates } }}$ Gene ${\mathop {\longrightarrow }\limits ^{\text { is upregulated by } }}$ Compound ${\mathop {\longrightarrow }\limits ^{\text { treats } }}$ Disease]
302	[Compound ${\mathop {\longrightarrow }\limits ^{\text { upregulates } }}$ Gene ${\mathop {\longrightarrow }\limits ^{\text { regulates } }}$ Gene ${\mathop {\longrightarrow }\limits ^{\text { is associated to } }}$ Disease]
259	[Compound ${\mathop {\longrightarrow }\limits ^{\text { treats } }}$ Disease ${\mathop {\longrightarrow }\limits ^{\text { associates } }}$ Gene ${\mathop {\longrightarrow }\limits ^{\text { is associated to } }}$ Disease]
152	[Compound ${\mathop {\longrightarrow }\limits ^{\text { upregulates } }}$ Gene ${\mathop {\longrightarrow }\limits ^{\text { is upregulated by } }}$ Anatomy ${\mathop {\longrightarrow }\limits ^{\text { is localized to } }}$ Disease]
137	[Compound ${\mathop {\longrightarrow }\limits ^{\text { upregulates } }}$ Gene ${\mathop {\longrightarrow }\limits ^{\text { is downregulated by } }}$ Anatomy ${\mathop {\longrightarrow }\limits ^{\text { is localized to } }}$ Disease]

Furthermore, actual applications of existing drugs are also published in^37^, so from there we identified that muscle cancer, colon cancer, and germ cell cancer are being treated with Epirubicin. With respect to muscle cancer, it is specified in DrugBank as soft tissue sarcoma^38^. In addition, Epirubicin has actually been proven to be evaluated for colorectal cancer^42^, being colon cancer predicted by AnyBURL-based methods. Similarly, the germ cell cancer inferred by AnyBURL maximum score, SAFRAN and XG4Repo, is the general name of a type of cancer that develops mainly in the ovary or testicle, being the ovarian cancer actually treated with Epirubicin^41^.

Moreover, in^37^ the finalised and active clinical trials can be found. For lymphatic system cancer also known as lymphoma, which is proposed as a candidate by SAFRAN and XG4Repo, different studies have been carried out focussing mainly on determining its effectiveness in combination with other drugs. For example, a recent study published in November 2022^40^ aims to evaluate the efficacy and safety of Camrelizumab combined with Epirubicin, Vincristine and Dacarbazine to treat patients with advanced classical Hodgkin’s lymphoma. They obtain an Objective Response Rate (ORR) of 100%, which means that 100% of the study patients had a partial and complete response within the study period.

Researchers are also studying the use of Epirubicin to treat melanoma^39^ in combination with other drugs.

For the rest of the diseases, current evidence of treatment has not been found in the literature. However, since these methods inferred these diseases, they could be potential candidates for diseases that could be treated with Epirubicin, providing a starting point for research.

#### Paclitaxel

Paclitaxel is a taxoid chemotherapeutic agent used as first-line and subsequent therapy for the treatment of advanced carcinoma of the ovary^44^. As shown in Table 5, all models except MINERVA have been able to predict ovarian cancer as a disease to be treated. In particular, XG4Repo proposes ovarian cancer as the first candidate.

**Table 5:** Top 10 diseases predicted by each model for the query “Paclitaxel treats disease”.

Disease	AnyBURL maxscore	AnyBURL noisy-OR	SAFRAN	MINERVA	XG4Repo
**Ovarian cancer**^44^	1	1	1		1
Pancreatic cancer^37^	3	3	4		2
Melanoma^37^	2	2	5		3
Stomach cancer^45^	4	4	3		4
Prostate cancer^46^	5	5	2		5
Hematologic cancer^47^		7	7	1, 2, 3, 8, 9, 10	6
Head and neck cancer^37^	6	6	6		7
Esophageal cancer^48^					8
Urinary bladder cancer^37^	9	9			9
Testicular cancer^37^	7		10		10
Hypertension^49^		8		6, 7	
Psoriasis^50^			8		
Colon cancer^51^	8	10			
Epilepsy				4	
Sarcoma	10				
Osteoporosis				5	

Each disease is predicted in a different position (rank) for different models. Diseases are ordered by XG4Repo results. Notice that in MINERVA, several paths can lead to the same node on different realisations. In bold, those diseases that are in the test set and therefore are true answers of the query.

In DrugBank^37^, we found that urinary bladder cancer, pancreatic cancer, testicular cancer, melanoma, head and neck cancer, and sarcoma are being treated with Paclitaxel, in combination with other drugs. Moreover, we have found in^37^ clinical trials that study the treatment of hematologic cancer^47^, stomach cancer^45^, prostate cancer^46^, psoriasis^50^, esophageal cancer^48^ and colon cancer^51^ with Paclitaxel. Paclitaxel has also been associated with the treatment of pulmonar hypertension^49^.

For the rest of the diseases, no evidence of treatment or clinical trials has been found yet.

#### Prednisone

Prednisone is a corticosteroid used to treat inflammation or immune-mediated reactions and to treat endocrine or neoplastic diseases^37^. Ulcerative colitis, hematologic cancer, atopic dermatitis, and chronic obstructive pulmonary disease can be treated with Prednisone and are included in the test set. As shown in Table 6, all models have been able to predict ulcerative colitis as a candidate and most of them hematologic cancer. XG4Repo has been able to identify chronic obstructive pulmonary disease as a candidate.

**Table 6:** Top 10 diseases predicted by each model for the query “Prednisone treats disease”.

Disease	AnyBURL maxscore	AnyBURL noisy-OR	SAFRAN	MINERVA	XG4Repo
**Ulcerative colitis**	2	1	1	1, 3, 4, 6, 7, 8, 9, 10	1
**Atopic dermatitis**					2
Allergic rhinitis^37^	1	2	2	5	3
**Chronic obstructive pulmonary disease**					4
**Hematologic cancer**	3	3	3		5
Amyotrophic Lateral Sclerosis^52^					6
Leprosy^53^					7
Bone cancer					8
Malaria					9
Primary biliary cholangitis					10
Osteoporosis^54^	10	7	7		
Lung cancer^55^		8	10		
Breast cancer^56^	4	5	4		
Hypertension^57^	8	4	5		
Coronary artery disease^58^	9	6	6		
Dilated cardiomyopathy			8		
Kidney cancer			9		
Epilepsy^59^				2	
Colon cancer	6				
Urinary bladder cancer^59^	7	9			
Testicular cancer^60^	5	10			

Each disease is predicted in a different position (rank) for different models. Diseases are ordered by XG4Repo results. Notice that in MINERVA, several paths can lead to the same node on different realisations. In bold, those diseases that are in the test set and therefore are true answers of the query.

Several models predict osteoporosis as a candidate; however, osteoporosis is a side effect of Prednisone^54^. As found in^18^, it is possible that metapaths find contraindications to the diseases, so it is always necessary to study the predictions before starting clinical trials.

We have found clinical trials using Prednisone for lung cancer^55^, breast cancer^56^, testicular germ cell cancer^60^, epilepsy in children^59^, leprosy^53^ and amyotrophic lateral sclerosis^52^.

In^37^, they propose Prednisone as a treatment for allergic rhinitis. In the case of hypertension, there are studies that relate the impact of Prednisone on this disease, but mainly in a negative way^57^. In^18^, they already found that some of the predictions made were contraindications to the disease, as the included relations are too general. The relation of Prednisone and coronary artery disease has also been studied^58^. The use of Prednisone to treat coronary artery disease has been studied in the past^61^, although it has not been used in general patients. This shows that our tool can make proposals similar to those made by a healthcare professional. Some studies also propose Prednisone to treat urinary bladder cancer^59^.

We can perform this analysis with any other drug present in the graph and obtain the rules and paths that support these predictions.

## Conclusion

In this work, we propose a general architecture to unify the process of graph-completion methods using paths. These methods are explainable, which is particularly relevant in the drug repurposing context. Moreover, they have good performance, which makes them trustworthy. We have analysed how some methods proposed in the literature fit our architecture and show that they are different approaches to complete the same stages of the process.

We have designed XG4Repo, a framework for drug repurposing using knowledge graphs that predict diseases that can be treated with a given compound. Along with the prediction, the model provides the rules that support the prediction and the importance of the rule. This step is necessary so that researchers can validate the prediction through the biological mechanism of action.

The results are presented for Hetionet, but the model can be trained on different knowledge graphs that include examples “compound treats disease”. Using other knowledge graphs could lead to different but relevant predictions. Training the model in larger knowledge graphs is the next step in this research.

We have included three use cases to show that the model is able to propose candidates similar to those proposed by humans. This is important because the objective of these tools is not to replace research, but to analyse large quantities of data in a short amount of time. Therefore, it is possible to accelerate the first stages of drug repurposing.

Regarding future lines that can extend this research, one of them is identifying and addressing potential biases in our model and/or dataset that could affect the accuracy of the drug prediction process. Another line could be analyzing the proposed explanations given by XG4Repo and assessing ways in which they can be improved. And finally, the performance of XG4Repo could be further assessed by making use of other repurposing databases, in order to detect ways in which it could be improved.

### Supplementary Information

Supplementary Information.

## Data Availability

The dataset used in this project is Hetionet^18^, which was developed within the Rephetio project and is publicly available in https://github.com/hetio/hetionet.

## Code availability

The code developed in this work is available in https://github.com/AnaJimBej/XG4Repo.
