# Supplementary material for: Explainable drug repurposing via path based knowledge graph completion
Source: Sci Rep. 2024 Jul 18;14:16587. doi: 10.1038/s41598-024-67163-x (PMC11258358; doi:10.1038/s41598-024-67163-x)
Supplement: Supplementary file 1 — Supplementary Information. [file 41598_2024_67163_MOESM1_ESM.pdf]

Supplementary Information: Explainable drug repurposing via path-based knowledge graph completion

Ana Jiménez [1,+], María José Merino [1,+], Juan Parras [1,*], and Santiago Zazo [1]

[1]Information Processing and Telecommunications Center, Universidad Politécnica de Madrid, ETSI Telecomunicación, Avda. Complutense, 30, 28040, Madrid, Spain
[*]j.parras@upm.es
[+]These authors contributed equally to this work

ABSTRACT

1 Mathematical framework formulation

We present a unified framework to understand several methods for graph completion based on path reasoning. Graphs are collections of objects (nodes) and the set of interactions (edges) between pairs of these objects[1]. Knowledge graphs ($\mathscr{G}$) are a particular type of multirelational graph where the information is defined by a set of existing triples, including a head node (h), a tail node (t^*) and a relation (r) that links them:

$$(h, r, t^*) \in \mathscr{G} \tag{1}$$

Drug repurposing on knowledge graphs can be seen as a task of link prediction, where we ask the graph which diseases a certain compound treats. We can understand the problem as a query that has to be solved by the graph. The query is composed of a compound (c) as the head, and the relation treats, $q = (c, treats)$. The answer to this query is a disease (d) that can be treated with the compound, which is the tail of the triple.

$$(c, treats, d^*) \in \mathscr{G} \tag{2}$$

The problem can be formulated in terms of the probability of success of the compound over the disease, where the objective is that the answer equals the tail of the triple $d = d^*$, and which is conditioned on the existing graph:

$$p(c, treats, d = d^* \mid \mathscr{G}) = p(d \mid \mathscr{G}, (c, treats)) = p(d \mid \mathscr{G}, q) \tag{3}$$

It is interesting to characterise a related conditional probability that depends on an additional variable μ that represents the selected set of policies under consideration, thus the set of rules or strategy that we follow to traverse the graph following paths toward a certain disease.

$$p(c, treats, d = d^* \mid \mathscr{G}, \mu) = p(d \mid \mathscr{G}, (c, treats), \mu) = p(d \mid \mathscr{G}, q, \mu) \tag{4}$$

The probability of a certain candidate disease d can be formulated as the softmax of a score function f_ω, which represents the good match between the expected response of the query and the candidate d:

$$p_\omega(d \mid \mathscr{G}, q, \mu) = \frac{\exp f_\omega(d; q, \mathscr{G}, \mu)}{\sum_{d' \in \mathscr{D}} \exp f_\omega(d'; q, \mathscr{G}, \mu)} \tag{5}$$

where $\mathscr{D}$ is the set of final nodes (diseases) that are reached when we run a set of trajectories in the graph following a certain policy μ.

The score is computed over the set of candidate answers and measures the plausibility of the node being the correct answer. It is a function of the candidate node being evaluated, the query, the graph, and the policy. The score function can be defined as:

$$f_\omega(d; q, \mathscr{G}, \mu) = \mathrm{AGG}(\{\psi_\omega(\mu_i)\, \mathrm{AGG}(\{\phi_\omega(d; \pi_i^n, q, \mathscr{G}, \mu_i)\}_{\pi_i^n})\}_{\mu_i \in \mu}) \tag{6}$$

and it is parameterised by ω. π_i^n represents paths generated according to the policy μ_i and AGG represents an aggregation operation such as a sum or a maximum. The first aggregation evaluates a set of policies, and the second aggregation evaluates the set of paths or trajectories generated by each policy. The functions ϕ_ω and ψ_ω can take different forms depending on the model and represent the importance of the path and the policy in the prediction. The quality of the policy μ_i is represented through ψ_ω, which is a trainable parameter that weights the importance of different policies. In models in which rules are used, ψ_ω measures the weight of the rule. The quality of the generated trajectories or paths is represented in the term ϕ_ω, which should have high values if the evaluated node (the final node of the trajectory) is the answer and low values otherwise.

1.1 Path generator

A policy generator is an element that generates policies. The policy is the strategy we use to traverse the graph. This generation process will be modelled as a random generator as follows:

$$p_\theta(\mu \mid \mathscr{G}, q) \tag{7}$$

whose distribution is parameterised by θ, where μ has to be understood as a set of policies.

$$\mu = \{\mu_i\}_{i=1}^{M} = \begin{bmatrix} \mu_1 & \mu_2 & \cdots & \mu_M \end{bmatrix} \tag{8}$$

Sampling any of these policies, which are themselves random variables, we can obtain trajectories:

$$\{\mu_i\} \sim p_\theta(\mu \mid \mathscr{G}, q) \rightarrow \{\pi_i^n\} \sim p_\theta(\pi \mid \mu_i) \tag{9}$$

where the trajectory π_i^n defines a sequence of nodes and the corresponding relations between them. An example of a path that proposes Epirubicin as a treatment for lung cancer is:

$$[\text{Epirubicine} \overset{\text{Upregulates}}{\longrightarrow} \text{Gene STK10} \overset{\text{Regulates}}{\longrightarrow} \text{Gene SOX2} \overset{\text{is associated with}}{\longrightarrow} \text{Lung cancer}]$$

In this project, we will also work with the concept of rule, which is another strategy to obtain paths or trajectories to traverse the graph in order to answer the query.

A rule is defined by the head and body following the structure[2]:

$$\text{head} \Rightarrow \text{body}$$

and the triplets take the form of:

$$\text{initial node type} \overset{\text{relation}}{\longrightarrow} \text{final node type}$$

The body of the rule gives a possible explanation for the relation represented by the head. Formally, the rules have the form of:

$$\text{Node type 0} \overset{\text{r}}{\longrightarrow} \text{Node type L} \Rightarrow [\text{Node type 0} \overset{r_1}{\longrightarrow} \text{Node type 1} \overset{r_2}{\longrightarrow} \text{Node type 2} \longrightarrow \cdots \overset{r_L}{\longrightarrow} \text{Node type L}]$$

where L is the length of the rule, node types represent the type of entity in the graph, for example, compound or disease, and r_l represents the relations or edges such as “treats” or “palliates”. An important property of these rules is composition, as it allows generating paths along the graph that can explain the head and make it more meaningful. Node type 0 and L, which are linked through the relation r, are also related through the sequence of relations r_1 to r_L.

For the objective of knowledge graph completion, missing triples are the head of the rule, and the body is used to find alternative paths between nodes that are plausible and equivalent to the relation in the head. In the drug repurposing case, the query is always “compound treats disease”.

A rule generator provides the sequence that defines the entity types and the relations that create the compositional effect. Defining by letter z the rules as latent variables, the rule generator will be represented by:

$$p_\theta(z \mid \mathscr{G}, q) \tag{10}$$

where each rule z_i is, in fact, an ordered sequence of edge types or relations (starting from e_0):

$$z_i = [r_1, r_2, ...r_L] \tag{11}$$

where the relation also defines the type of nodes, as a relation can only exist between specific node types, for example, the relation “treats” can only exist between a compound and a disease. Each realisation of z represents a different sequence z_i that can be translated into a trajectory.

$$z = \{z_i\}_{i=1}^{M} = \begin{bmatrix} z_1 & z_2 & \cdots & z_M \end{bmatrix} \tag{12}$$

$$\{z_i\} \sim p_\theta(z \mid G, q) \to \{\pi_i^n\} \sim p_\theta(\pi \mid z_i) \tag{13}$$

As the rule is defined by the relation, the same rule z_i generates multiple trajectories that differ at the nodes. Applying the rule:

$$\text{Compound} \xrightarrow{\text{treats}} \text{Disease} \Rightarrow [\text{Compound} \xrightarrow{\text{causes}} \text{Side effect} \xrightarrow{\text{is caused by}} \text{Compound} \xrightarrow{\text{treats}} \text{Disease}]$$

the query can be solved because a compound "Lapatinib" causes nausea like "Captopri" which treats hypertension or because "Lapatinib" causes insomnia like "Bepridil" which treats hypertension. The same rule generates two trajectories. Furthermore, the same generator can generate another rule:

$$\text{Compound} \xrightarrow{\text{treats}} \text{Disease} \Rightarrow [\text{Compound} \xrightarrow{\text{downregulates}} \text{Gene} \xrightarrow{\text{is associated to}} \text{Disease} \xrightarrow{\text{associates}} \text{Gene} \xrightarrow{\text{is associated to}} \text{Disease}]$$

which also relates compounds with the diseases they treat.

In the same way, the policy represents sequences of nodes and relations in the form of trajectories or paths.

Taking into account the concepts of policy generator and score function, the probability of the candidate for repurposing can be parameterised by θ and ω.

$$p(d \mid \mathscr{G}, q) \to p_{\omega,\theta}(d \mid \mathscr{G}, q) \tag{14}$$

This expression can be decomposed into two processes: path generation and reasoning prediction. The objective of the path generator (parameterised by θ) is to obtain the policy/rules that models the problem, and the reasoning predictor (parameterised by ω) uses those paths to answer queries.

$$p_{\omega,\theta}(d \mid \mathscr{G}, q) = \sum_{\mu} p_\omega(d \mid \mathscr{G}, q, \mu) p_\theta(\mu \mid \mathscr{G}, q) \tag{15}$$

$$p_{\omega,\theta}(d \mid \mathscr{G}, q) = \sum_{z} p_\omega(d \mid \mathscr{G}, q, z) p_\theta(z \mid \mathscr{G}, q) \tag{16}$$

The objective of the graph completion task, which is to predict correct answers, is now modelled by a path generator that has to be optimised to provide the best trajectories and a reasoning predictor that gives the likelihood of the answer d conditioned on a latent set of policies μ or rules z, the query q, and the knowledge graph. Then, the problem can be structured in two steps:

- To evaluate the predictor using some rules/paths for training.
- To generate (good) paths. The method of obtaining policies differentiates most algorithms.

We present in Figure S1 the general scheme that the algorithms described are going to follow to solve the problem of answering queries from the graph.

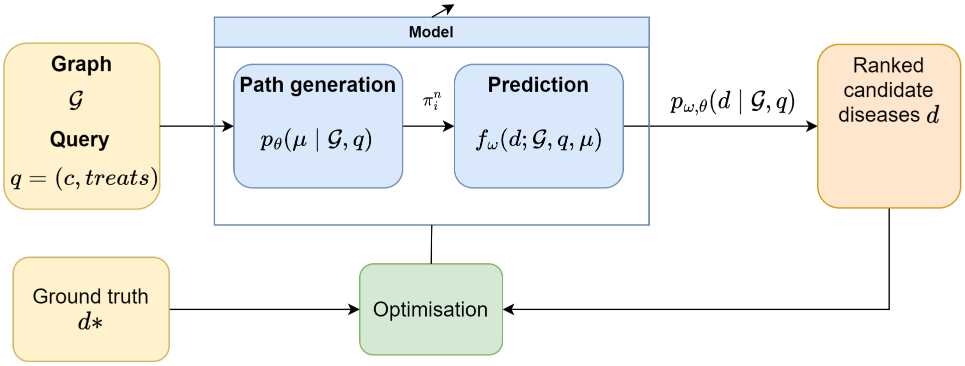

Figure S1. Representation of the graph completion process using path reasoning. Both steps of the process can be distinguished: the reasoning predictor based on the score function and the policy generator used to generate trajectories or paths for the prediction. The representation is the same in the case rules z are used instead of policies μ.

1.2 Fixed policy

There are several ways to generate paths and the most simple one is to assume that the generator is fixed. We can generate paths following different principles, for example random walks, Breadth First Search (BFS) or Depth First Search (DFS). Using these fixed methods, we can obtain paths to traverse the graph from a starting compound node to a final disease node.

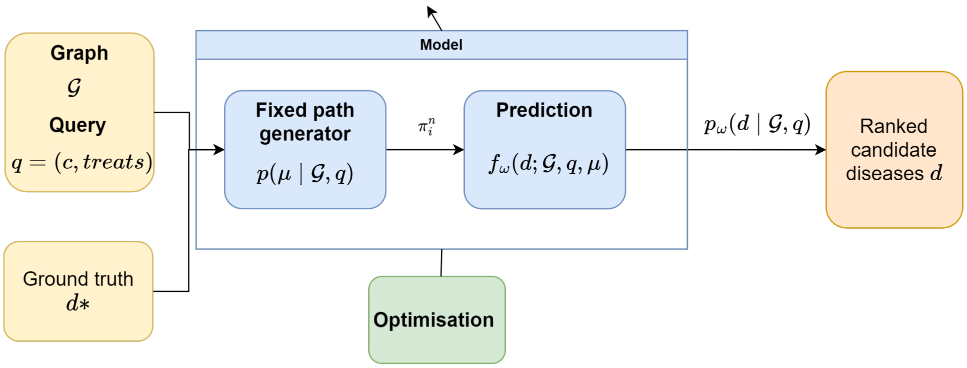

Figure S2. Architecture of the path based model for drug repurposing considering fixed path generation. The strategy to obtain the paths is always the same and independent of the prediction results. We represent the expression of policy μ for simplicity, but rule generators also fit this description.

The paths π_i^n obtained with the generator propose a set of candidate diseases that have to be ranked according to the scoring function $f_\omega(d; \mathcal{G}, q, \mu)$. With that score function, we can obtain the probability that a certain compound treats the candidate disease. The parameters of the score function can be optimised to maximise the probability of true treatments.

AnyBURL[3], and therefore SAFRAN[4], are models that use this workflow as shown in Figure S3 to generate rules. In this case, the fixed path generator consists of a path sampler and a rule generator. Rules are generalised from paths sampled using random walks. Only rules with a score higher than a threshold are kept. The rules are then used for prediction, using different score functions based on the confidence of the rules[4,5].

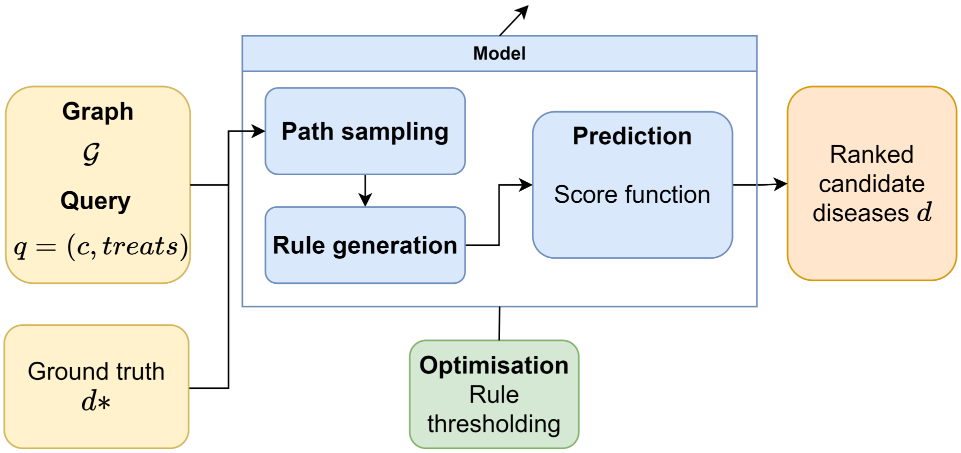

Figure S3. Description of AnyBURL based on our architecture. In this case, the input of the model is the whole graph (training set) including the ground truth. Paths are sampled based on random walk, and they are used to generate rules using a bottom-up approach. Then the confidence of the rule is computed, so only the rules with a confidence higher than a threshold are used for prediction. Rules are applied to the graph to obtain predictions that are ranked using the confidence of the rule.

1.3 Reinforcement learning

The next step is to use generators that can be updated to find the best paths between the compounds and the corresponding diseases. Some methods use reinforcement learning to model the trajectory on the graph as a Markov Decision Process. The objective of the reinforcement learning problem is to find the optimal policy to answer the queries. Starting from the head node, the agent learns to walk to the tail node, choosing intermediate nodes step by step, taking into account the path history.

In the drug repurposing context, the environment is the graph, and the possible actions are all the links the agent can choose from a certain node to the next. The objective of the agent is to move from a compound node to a disease node that is linked through the relations "treats". The state includes all nodes and relationships travelled through to the current node, so the next action depends on the whole path. For that reason, it has to be modelled with a structure with memory such as a recurrent neural network.

The trajectory π can be modeled as:

$$p_\theta(\pi \mid \mathscr{G}, q) = p_\theta(a_1, a_2, ..., a_L \mid \mathscr{G}, q) = \prod_{l=1}^{L} p_\theta(a_{S_l} \mid S_l) \tag{17}$$

where S_l is the state and a_{S_l} is the action taken at state S_l. The state includes information about the query, the initial entity (head of the query), the relation, and the previous actions or relations selected. The graph is also represented in the state since the choice of actions represents a path through the graph. The selected action a_{S_l} depends on the history states S_l up to step l, which is the current state.

Reinforcement learning methods also require the definition of the reward function that indicates whether the actions taken are good or not. In the most simple case,

$$\begin{cases} R(S_L \mid q) = 1 & \text{if } d = d^* \\ R(S_L \mid q) = 0 & \text{if } d \neq d^* \end{cases} \tag{18}$$

where d is the final node at step L, so the reward is positive if the agent reaches the final target at step L and zero if not. Gradient-based optimisation techniques are applicable if we have N trajectories for policy μ_θ (considering that here we have a single policy, as we are working on-policy).

For models that are optimised using policy search, the objective function of the policy gradient algorithm is the expected return of the policy.

$$\mathbb{E}_{\pi_\theta^n \sim \mu_\theta}\left[R(S_L \mid q)\right] \tag{19}$$

A widely used estimator for this objective function is the following one, based on trajectories sampled from the policy:

$$\mathbb{E}_{\pi_\theta^n \sim \mu_\theta}\left[R(S_L \mid q)\right] \approx \frac{1}{N}\sum_{n=1}^{N} R(S_L^n \mid q)\, p_\theta(\pi_\theta^n \mid \mathscr{G}, q) = \frac{1}{N}\sum_{n=1}^{N} R(S_L^n \mid q) \prod_{l=1}^{L} p_\theta(a_{S_l^n} \mid S_l^n) \tag{20}$$

Replacing the probability by the logarithm of the probability, the objective function that needs to be maximised is the following:

$$\frac{1}{N}\sum_{n=1}^{N} R(S_L^n \mid q) \sum_{l=1}^{L} \log p_\theta(a_{S_l^n} \mid S_l^n) \tag{21}$$

This is the objective function for one query q, to train the model, we average over the whole training set.

Policy search is an algorithm whose objective focuses on maximising the reward to obtain the parameters of the policy generator. We are working on policy, so only one policy is taken into account; therefore, the score function has to be particularised for that case.

$$f_\theta(d; q, \mathscr{G}, \mu_\theta) = \sum_{\pi_\theta^k} \phi_\theta\left(d; \pi_\theta^k, q, \mathscr{G}, \mu_\theta\right) = \sum_{\pi_\theta^k}\sum_{l=1}^{L} \phi_{\theta,l}\left(a_{S_l^n}; \pi_\theta^k, q, \mathscr{G}, \mu_\theta\right) \tag{22}$$

where $k \in \mathbb{K}$ represents all the trajectories that have d as the final node. We use parameters θ as the predictor is part of the policy generator, the predictions are used to generate paths choosing based on the probability step by step. The probability of each of these nodes can be derived from Equation 21, where in this equation (Equation 21) the final nodes of the N trajectories are taken into account $d \in \mathscr{D}$:

$$p_\theta(d \mid \mathscr{G}, q, \mu_\theta) = \frac{1}{K}\sum_{\pi_\theta^k} R(S_L^k \mid q) \sum_{l=1}^{L} \log p_\theta(a_{S_l^k} \mid S_l^k) \tag{23}$$

and related to Equation 22 since the probability is the softmax of the score function as represented in Equation 5.

The algorithm can be summarised in the following scheme:

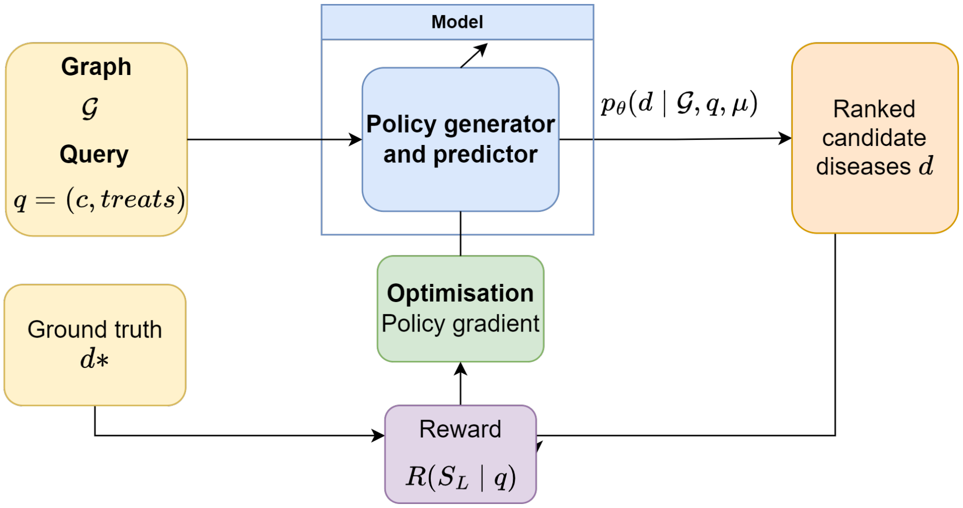

Figure S4. Description reinforcement learning model for path reasoning, in particular, MINERVA[6]. The core of the algorithm is the policy generator, which is trained to obtain the best policy through the reward using policy search. Paths are sampled from the generator to obtain candidate diseases which are ranked according to the path that proposes them.

The algorithm presents two main blocks: the policy generator combined with reasoning predictor and the optimisation block based on policy gradient. When the current policy is sampled μ_θ, the trajectory is generated conditioned on the query and the graph, and a final node is reached. In this case, the reasoning predictor is included in the policy generator, as the action or relation selected at each step depends on the score function and the result of the predictor. Once the agent reaches the final node, the reward is observed and used to optimise the policy using policy gradient and the objective function presented in Equation 20 and Equation 21.

MINERVA[6] is a reinforcement learning agent optimised using this policy search formulation, in particular, REINFORCE. Other models have included modifications in MINERVA, such as PoLo[7], which includes more terms in the reward, so paths that follow metapaths that are known to be useful have a higher reward, or DIVINE[8], which adds generative adversarial reasoner to the process.

1.4 Rule based path reasoning

Reasoning based on reinforcement learning has the problem that the action space is large and the reward is sparse, as few paths lead to the correct answer and a positive reward. There are other strategies, such as those that use trainable rule generators to obtain paths for prediction.

Drug repurposing based on rules consists of a rule generator and a reasoning predictor with logic rules, which are independent and trained simultaneously to improve each other[9]. The reasoning predictor uses the logic rules provided by the rule generator to answer queries, providing an effective reward to train the rule generator, which helps significantly reduce the search space.

Based on a query $q = (c, treats, d^*)$, the probability of the answer conditioned on the existing knowledge graph $p(d = d^* \mid G, q) = p(d \mid \mathcal{G}, q)$ is modelled using a set of logic rules z, treated as a latent variable that must be inferred.

$$p_{\omega,\theta}(d \mid \mathcal{G}, q) = \sum_z p_\omega(d \mid \mathcal{G}, q, z) p_\theta(z \mid \mathcal{G}, q) \tag{24}$$

The rule generator defines a prior distribution on the logic rules for each query, $p_\theta(z \mid q)$, which is parameterised by a recurrent neural network. The probability of the rule generator that generates rules of length L can be defined as:

$$\begin{aligned} p_\theta(z \mid \mathcal{G}, q) &= p_\theta(r_1, r_2, \ldots r_L \mid \mathcal{G}, q) = \\ &= p_\theta(r_1 \mid e_0) p_\theta(r_2 \mid e_0, r_1) \ldots p_\theta(r_L \mid e_0, r_1, r_2, \ldots r_L) = \\ &= \prod_{l=1}^{L} p_\theta(r_l \mid S_l) \end{aligned} \tag{25}$$

where r_i is the relation at step i, and S_l is defined as the state and includes information about the query, the initial entity (head of the query), the relation, and the previous relations selected. The graph is also represented in the state, since the choice of actions represents a path through the graph. This rule generator is equivalent to the policy generator defined previously.

The reasoning predictor computes the likelihood of the answer conditioned on the logic rules and the existing knowledge graph $\mathscr{G}$, $p_\omega(d \mid \mathscr{G}, q, z)$. At each training iteration, a few logic rules are sampled from the generator, which are fed into the reasoning predictor to test these rules for prediction. The distribution $p(d \mid \mathscr{G}, q)$ can be calculated according to Equation 15 as:

$$p_{w,\theta}(d \mid \mathscr{G}, q) = \sum_z p_w(d \mid \mathscr{G}, q, z) p_\theta(z \mid q) = \mathbb{E}_{p_\theta(z|q)} \left[p_w(d \mid \mathscr{G}, q, z) \right] \tag{26}$$

which is the objective function that has to be optimised by the whole model. This task is divided as the generator and predictor use different optimisation algorithms, but both contribute to a common goal.

Sampling the rule generator, we obtain the rules z_i which define the metapaths. The particularisation of the rule on the graph gives what is called the path, the sequence of nodes and relations, obtained applying the rule to a certain query. This path is what we consider to be a trajectory π_i^n.

RNNLogic[9] implements this architecture. The model includes a rule generator and a reasoning predictor that applies the rules to propose candidate predictions. The rule generator uses a recurrent neural network to generate a set of logic rules conditioned on the query, which are given to the reasoning predictor for query answering.

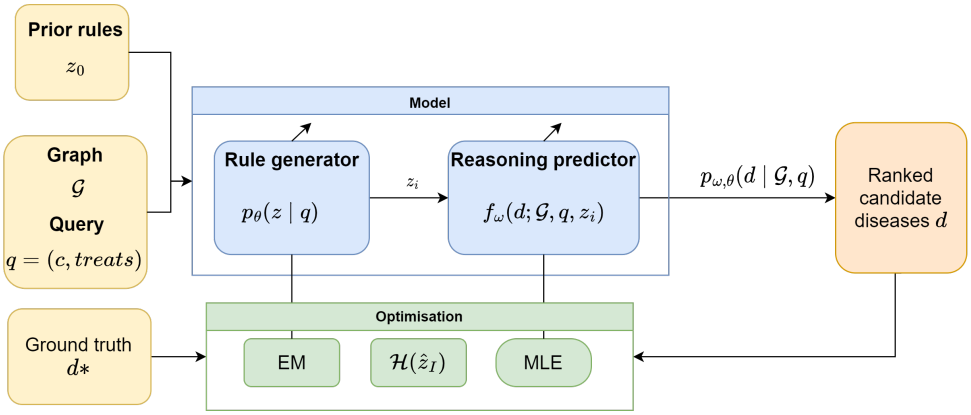

Figure S5. Process followed by RNNLogic for the graph completion task. Two main blocks, rule generator and reasoning predictor enhance each other to predict the correct answers for the queries. In addition to the graph and the query, there is another input which is a set of prior rules to initialize the generator. The model consists of a rule generator and a reasoning predictor. A set of rules is sampled and used for prediction. During training, the predictor is updated using maximum likelihood estimation (MLE). Combining information of the generation and the prediction, a score for each rule $\mathscr{H}(z_i)$ is computed and it is using during the training of the generator which is based on expectation maximisation.

The optimisation process is based on the EM algorithm for the rule generator and the maximum likelihood estimation principles (MLE) for the reasoning predictor, as shown in Figure S5. In each iteration, a set of rules z_i is sampled from the generator and used to update the predictor. Based on the result of the predictor, a set of high-quality rules is identified via posterior inference, taking into account the prior of the generator and the likelihood of the reasoning predictor.

The criteria for selecting the set of high-quality rules is the posterior distribution of each subset of logic rules. The authors use an approximation of the posterior probability $\mathscr{H}(\hat{z}_I)$, as the exact calculation is nontrivial because of its intractable partition function. Intuitively, the score $\mathscr{H}(\hat{z}_I)$ represents the quality of the rule taking into account the generator and the predictor. The scores should be high for rules that lead to true predictions according to the predictor. Moreover, the score should consider the prior probability of the rule given by the generator.

This set of high-quality rules is used to update the generator. This model requires a set of pre-computed rules and their corresponding prior scores to start the process. Different models can be used to obtain the initial set of rules based on the training data from the graph, for example AnyBURL[3].

The objective is to jointly train the rule generator and the reasoning predictor to maximise the logarithmic likelihood of the training data conditioned on the graph and the query.

$$\log p_{w,\theta}(d \mid \mathscr{G}, q) = \log \mathbb{E}_{p_\theta(z|q)} \left[p_w(d \mid \mathscr{G}, q, z) \right] \tag{27}$$

where the generator is parameterised by θ and the predictor by w. The graph is represented by $\mathscr{G}$, the query by q and the set of rules by z.

As there is an expectation operation related to the generator, a sample $\hat{z} \sim p_\theta(z \mid q)$ is used to approximate the objective function for each training instance as:

$$\log \mathbb{E}_{p_\theta(z|q)}[p_w(d \mid \mathscr{G}, q, z)] \approx \log p_w(d \mid \mathscr{G}, q, \hat{z}) \tag{28}$$

which represents the function that has to be maximised.

The set of rules generated $\hat{z}$ can be used by the predictor to find paths in the graph $\mathscr{G}$ leading to different candidate answers. Each candidate answer has a score:

$$f_\omega(d; q, \mathscr{G}, z) = \sum_{z_i \in \hat{z}} \psi_\omega(z_i) \sum_{\pi_i^n} \phi_\omega(e, \pi_i^n, \mathscr{G}, q, z_i) \tag{29}$$

where ψ_ω is the score of the rule z_i, which is a trainable parameter, and ϕ_ω is the score of the path π_i^n, taking into account the final node d, the trajectory through the graph, and the query. The path score should be high if the final node is the correct answer node and low otherwise. Once we have the score for each candidate answer, we can further define the probability that the answer d of query q is correct using a softmax function as in Equation 5. Then, the parameters ω are updated to maximise the log-likelihood of the correct answer.

To optimise the generator $p_\theta(z \mid q)$, a set of high-quality rules $\hat{z}_I$ is selected according to $\mathscr{H}(\hat{z}_I)$. For each data instance, the set of rules $\hat{z}_I$ is treated as part of the training data, and the generator is updated by maximising the logarithmic likelihood of $\hat{z}_I$. Moreover, $\mathscr{H}(\hat{z}_I)$ has information on the quality of the rules, so it can also be included in the generator optimisation in the form of weights of each rule:

$$\mathscr{H}(\hat{z}_I) \log p_\theta(\hat{z}_I \mid q) = \sum_{z_i \in \hat{z}_I} \mathscr{H}(\hat{z}_I) \sum_{l=1}^{L} \log p_\theta(r_{l^i} \mid S_l) \tag{30}$$

where r_{l^i} is the relation at step l of the rule i.

The optimisation of the rule generator in the rule-based architecture has strong connections with policy search algorithm used in reinforcement learning. The objective function of the rule generator is:

$$\sum_{z_i \in \hat{z}_I} \mathscr{H}(\hat{z}_I) \sum_{l=1}^{L} \log p_\theta(r_{l^i} \mid S_l) \tag{31}$$

considering one training sample and a set of high quality sampled rules $\hat{z}_I$. These rules generate a set of trajectories in the same way as sampled paths in reinforcement learning. The objective function in this case is defined as:

$$\sum_{n=1}^{N} R\left(S_L^N \mid q\right) \sum_{l=1}^{L} \log p_\theta(a_{S_l^n} \mid S_l^n) \tag{32}$$

so both functions are equivalent if we consider the posterior score $\mathscr{H}(\hat{z}_I)$ of rule-based as a reward. Reinforcement learning has the limitation that the reward is always set to 1 if the correct answer is reached and to 0 otherwise. The rule-based score includes more information as it combines the prior score of the rule with the result of the prediction.

Regarding the trajectories, there are also some differences. In reinforcement learning, L trajectories are generated in the form of multiple trials of the same query and the same policy. In rule-based models, the trajectories are generated by a set of rules $\hat{z}_I$. These rules are a subset of the rules used for prediction to select the best rules for optimisation. Both approaches coincide if the score function of rule-based models (which behaves as a reward) is set to 1 for the top rules selected from the posterior and to 0 otherwise.

References

1. Hamilton, W. L. *Graph Representation Learning* (Springer International Publishing, 2020).
2. Ji, S., Pan, S., Cambria, E., Marttinen, P. & Yu, P. S. A Survey on Knowledge Graphs: Representation, Acquisition, and Applications. *IEEE Transactions on Neural Networks Learn. Syst.* **33**, 494–514 (2022).
3. Meilicke, C., Chekol, M. W., Ruffinelli, D. & Stuckenschmidt, H. Anytime bottom-up rule learning for knowledge graph completion. *IJCAI Int. Jt. Conf. on Artif. Intell.* **2019-August**, 3137–3143 (2019).
4. Ott, S., Meilicke, C. & Samwald, M. Safran: An interpretable, rule-based link prediction method outperforming embedding models. *arXiv preprint arXiv:2109.08002* (2021).

5. Meilicke, C., Chekol, M. W., Fink, M. & Stuckenschmidt, H. Reinforced anytime bottom up rule learning for knowledge graph completion. *arXiv preprint arXiv:2004.04412* (2020).

6. Das, R. *et al.* Go for a walk and arrive at the answer: Reasoning over paths in knowledge bases using reinforcement learning. *arXiv preprint arXiv:1711.05851* (2017).

7. Liu, Y., Hildebrandt, M., Joblin, M., Ringsquandl, M. & Tresp, V. Integrating logical rules into neural multi-hop reasoning for drug repurposing. *arXiv preprint arXiv:2007.05292* (2020).

8. Li, R. & Cheng, X. Divine: a generative adversarial imitation learning framework for knowledge graph reasoning. In *Proceedings of the 2019 Conference on Empirical Methods in Natural Language Processing and the 9th International Joint Conference on Natural Language Processing (EMNLP-IJCNLP)*, 2642–2651 (2019).

9. Qu, M., Chen, J., Xhonneux, L.-P., Bengio, Y. & Tang, J. Rnnlogic: Learning logic rules for reasoning on knowledge graphs. In *International Conference on Learning Representations* (2021).
